# Crystal structure of 3,4′-diphenyl-3′-*p*-tolyl-4′*H*-spiro­[indan-2,5′-[1,2]oxazol]-1-one

**DOI:** 10.1107/S2056989015019581

**Published:** 2015-10-24

**Authors:** Asmae Mahfoud, Ghali Al Houari, Mohamed El Yazidi, Mohamed Saadi, Lahcen El Ammari

**Affiliations:** a Laboratoire de Chimie Organique, Faculté des Sciences Dhar el Mahraz, Université Sidi Mohammed Ben Abdellah, BP 1796 Atlas, 30000 Fès, Morocco; b Laboratoire de Chimie du Solide Appliquée, Faculté des Sciences, Université Mohammed V, Avenue Ibn Battouta, BP 1014, Rabat, Morocco

**Keywords:** crystal structure, hydrogen-bonding, 1,3-dipolar cyclo­addition reaction

## Abstract

In the title compound, C_30_H_23_NO_2_, the five-membered rings are both in envelope conformations with the same spiro C atom as the flap. The benzene ring and the two phenyl rings are inclined to the mean plane of the indene ring system by 83.98 (8), 81.46 (8) and 72.31 (7)°. In the crystal, mol­ecules are linked by pairs of C—H⋯O hydrogen bonds into inversion dimers. The dimers are further connected by C—H⋯N inter­actions, forming layers parallel to (10-1).

## Related literature   

For general background to 1,3-dipolar cyclo­addition reactions, see: Al Houari *et al.* (2008 ▸, 2010 ▸). For a related structure, see: Akhazzane *et al.* (2010 ▸).
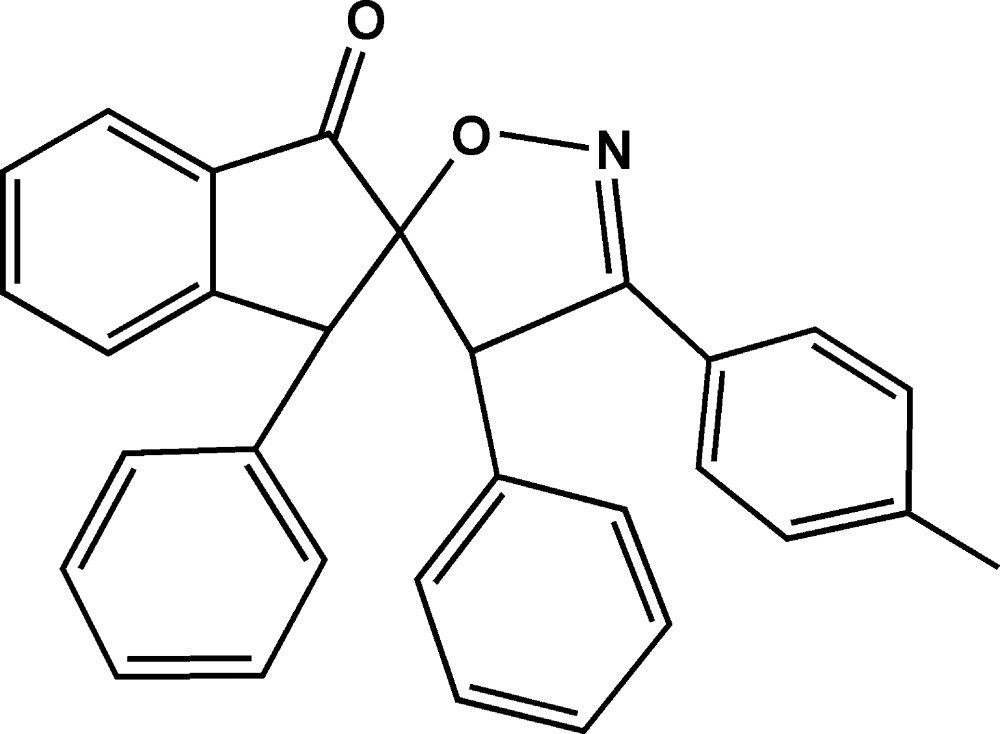


## Experimental   

### Crystal data  

C_30_H_23_NO_2_
*M* *_r_* = 429.49
Monoclinic, 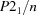

*a* = 9.7381 (7) Å
*b* = 20.5072 (14) Å
*c* = 11.8261 (8) Å
β = 102.836 (2)°
*V* = 2302.7 (3) Å^3^
*Z* = 4
Mo *K*α radiation
μ = 0.08 mm^−1^
*T* = 296 K
0.42 × 0.31 × 0.26 mm

### Data collection  

Bruker X8 APEX diffractometer
38803 measured reflections
5942 independent reflections
3783 reflections with *I* > 2σ(*I*)
*R*_int_ = 0.042

### Refinement  

*R*[*F* ^2^ > 2σ(*F* ^2^)] = 0.049
*wR*(*F* ^2^) = 0.150
*S* = 1.03
5942 reflections
298 parameters
H-atom parameters constrained
Δρ_max_ = 0.21 e Å^−3^
Δρ_min_ = −0.21 e Å^−3^

### 

Data collection: *APEX2* (Bruker, 2009 ▸); cell refinement: *SAINT* (Bruker, 2009 ▸); data reduction: *SAINT*; program(s) used to solve structure: *SHELXT* (Sheldrick, 2015*a* ▸); program(s) used to refine structure: *SHELXL2014* (Sheldrick, 2015*b* ▸); molecular graphics: *ORTEP-3 for Windows* (Farrugia, 2012 ▸); software used to prepare material for publication: *PLATON* (Spek, 2009 ▸) and *publCIF* (Westrip, 2010 ▸).

## Supplementary Material

Crystal structure: contains datablock(s) I. DOI: 10.1107/S2056989015019581/is5427sup1.cif

Structure factors: contains datablock(s) I. DOI: 10.1107/S2056989015019581/is5427Isup2.hkl

Supporting information file. DOI: 10.1107/S2056989015019581/is5427Isup3.cml

**Fig. 1.**
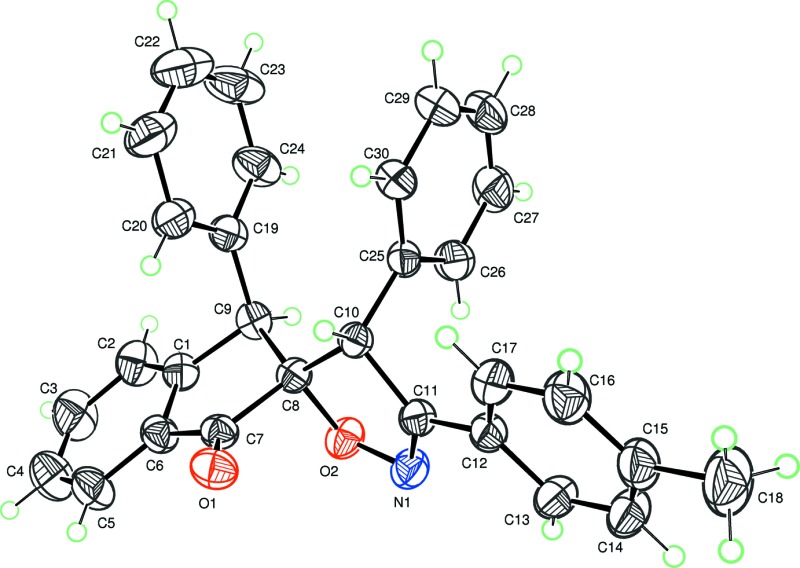
The molecular structure of the title compound with the atom-labelling scheme. Displacement ellipsoids are drawn at the 50% probability level. H atoms are represented as small circles.

. DOI: 10.1107/S2056989015019581/is5427fig1.tif
The mol­ecular structure of the title compound with the atom-labelling scheme. Displacement ellipsoids are drawn at the 50% probability level. H atoms are represented as small circles.

**Fig. 2.**
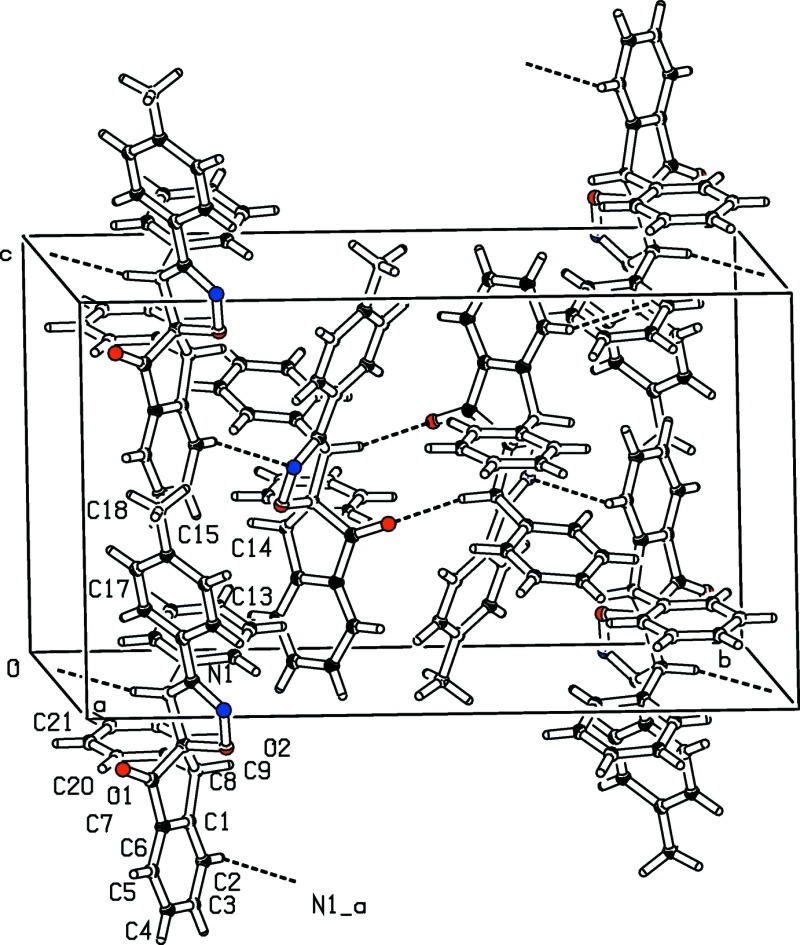
: Partial crystal packing for the title compound showing molecules linked by hydrogen bonds as dashed lines.

. DOI: 10.1107/S2056989015019581/is5427fig2.tif
Partial crystal packing for the title compound showing mol­ecules linked by hydrogen bonds as dashed lines.

CCDC reference: 1431561

Additional supporting information:  crystallographic information; 3D view; checkCIF report

## Figures and Tables

**Table 1:** Hydrogen-bond geometry (, )

*D*H*A*	*D*H	H*A*	*D* *A*	*D*H*A*
C10H10O1^i^	0.98	2.47	3.4169(18)	163
C2H2N1^ii^	0.93	2.56	3.280(2)	135

Symmetry codes: (i) 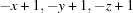; (ii) 
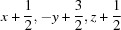
.

## supplementary crystallographic information

### S1. Comment

In this paper we studied the stereochemistry in the reaction of *p*-tolylnitriloxide with (2*E*)-2-benzylidene-3-phenyl-2,3-dihydro-1*H*-inden-1-one. The X-Ray crystal study shows that the cabonyl group is in the position 5 of the isoxazoline. We also found that the phenyl group imposes an exclusive anti approach of the dipole. This stereochemistry is due to steric effects (Al Houari *et al.*, 2008, 2010; Akhazzane *et al.*, 2010).

The molecule of the title compound is formed by two fused five- and six-membered rings linked to a phenyl ring and to a five-membered ring which is connected to a phenyl ring and a toluene cycle (Fig. 1). The two five-membered rings (C1/C6–C9) and (N1/O2/C8/C10/C11) adopt envelope conformations on atom C8 as indicated by the total puckering amplitude Q2 = 0.256 (2) Å and spherical polar angle φ2 = 290.0 (4)°, and Q2 = 0.2496 (2) Å and φ2 = 320.0 (3)°. The mean plane through the indene ring (C1–C9) is nearly perpendicular to the benzene and phenyl rings (C12–C17, C19–C24 and C25–C30), making dihedral angles of 83.98 (8), 81.46 (8) and 72.31 (7)° with them. In the crystal, molecules are linked by a pair of C10—H10···O1 hydrogen bonds into an inversion dimer. The dimers are further connected by a C2—H2···N1 interaction (Fig. 2 and Table 1).

### S2. Experimental

In a 100 ml flask, we dissolve 2 mmoles of (2*E*)-2-benzylidene-3-phenyl-2,3-dihydro-1*H*-inden-1-one and 2.4 mmoles of *p*-tolyloxime in 20 ml of chloroform. The mixture is cooled to 273 K under magnetic stirring in an ice bath. Then 15 ml of bleach (NaOCl) at 291 K (Chlorometric degree) is added in small doses without exceeding 278 K. The mixture is left under magnetic stirring for 16 h at room temperature, then washed with water until the pH is neutral and dried on sodium sulfate. The solvent is evaporated with a rotating evaporator and the oily residue is dissolved in ethanol. The precipitated compound is then recrystallized in ethanol.

### S3. Refinement

H atoms were located in a difference map and treated as riding with C—H = 0.96, 0.98 and 0.93 Å for methyl, methine and aromatic, respectively. *U*_iso_(H) values were set at 1.2*U*_eq_(C) for methine and aromatic, and 1.5*U*_eq_(C) for methyl. The reflection (0 1 1) affected by the beamstop was removed during refinement.

### Crystal data

**Table:**

C_30_H_23_NO_2_	*F*(000) = 904
*M**_r_* = 429.49	*D*_x_ = 1.239 Mg m^−^^3^
Monoclinic, *P*2_1_/*n*	Mo *K*α radiation, λ = 0.71073 Å
*a* = 9.7381 (7) Å	Cell parameters from 5942 reflections
*b* = 20.5072 (14) Å	θ = 2.4–28.7°
*c* = 11.8261 (8) Å	µ = 0.08 mm^−^^1^
β = 102.836 (2)°	*T* = 296 K
*V* = 2302.7 (3) Å^3^	Block, colourless
*Z* = 4	0.42 × 0.31 × 0.26 mm

### Data collection

**Table:**

Bruker X8 APEX diffractometer	3783 reflections with *I* > 2σ(*I*)
Radiation source: fine-focus sealed tube	*R*_int_ = 0.042
Graphite monochromator	θ_max_ = 28.7°, θ_min_ = 2.4°
φ and ω scans	*h* = −12→13
38803 measured reflections	*k* = −27→27
5942 independent reflections	*l* = −15→15

### Refinement

**Table:**

Refinement on *F*^2^	0 restraints
Least-squares matrix: full	Hydrogen site location: inferred from neighbouring sites
*R*[*F*^2^ > 2σ(*F*^2^)] = 0.049	H-atom parameters constrained
*wR*(*F*^2^) = 0.150	*w* = 1/[σ^2^(*F*_o_^2^) + (0.0706*P*)^2^ + 0.3191*P*] where *P* = (*F*_o_^2^ + 2*F*_c_^2^)/3
*S* = 1.03	(Δ/σ)_max_ < 0.001
5942 reflections	Δρ_max_ = 0.21 e Å^−^^3^
298 parameters	Δρ_min_ = −0.21 e Å^−^^3^

### Special details

**Table:**

Geometry. All e.s.d.'s (except the e.s.d. in the dihedral angle between two l.s. planes) are estimated using the full covariance matrix. The cell e.s.d.'s are taken into account individually in the estimation of e.s.d.'s in distances, angles and torsion angles; correlations between e.s.d.'s in cell parameters are only used when they are defined by crystal symmetry. An approximate (isotropic) treatment of cell e.s.d.'s is used for estimating e.s.d.'s involving l.s. planes.

### Fractional atomic coordinates and isotropic or equivalent isotropic displacement parameters (Å^2^)

**Table:**

	*x*	*y*	*z*	*U*_iso_*/*U*_eq_	
C1	0.63361 (17)	0.65004 (7)	0.77155 (13)	0.0434 (4)	
C2	0.7018 (2)	0.67536 (9)	0.87761 (15)	0.0615 (5)	
H2	0.7760	0.7046	0.8828	0.074*	
C3	0.6574 (3)	0.65632 (12)	0.97544 (16)	0.0798 (6)	
H3	0.7009	0.6739	1.0469	0.096*	
C4	0.5499 (3)	0.61194 (12)	0.96970 (17)	0.0823 (7)	
H4	0.5226	0.5999	1.0372	0.099*	
C5	0.4821 (2)	0.58502 (10)	0.86457 (16)	0.0646 (5)	
H5	0.4103	0.5546	0.8602	0.078*	
C6	0.52553 (17)	0.60528 (7)	0.76583 (13)	0.0451 (4)	
C7	0.47174 (15)	0.58614 (7)	0.64401 (12)	0.0391 (3)	
C8	0.52976 (15)	0.63745 (6)	0.57117 (12)	0.0363 (3)	
C9	0.66333 (16)	0.66398 (7)	0.65309 (12)	0.0406 (3)	
H9	0.6690	0.7112	0.6424	0.049*	
C10	0.52416 (15)	0.61711 (6)	0.44621 (11)	0.0355 (3)	
H10	0.5327	0.5696	0.4410	0.043*	
C11	0.37483 (16)	0.63844 (7)	0.39210 (13)	0.0397 (3)	
C12	0.29663 (16)	0.62078 (7)	0.27526 (13)	0.0419 (3)	
C13	0.17247 (17)	0.65406 (9)	0.22408 (15)	0.0541 (4)	
H13	0.1390	0.6870	0.2649	0.065*	
C14	0.1000 (2)	0.63836 (10)	0.11407 (16)	0.0629 (5)	
H14	0.0188	0.6615	0.0811	0.075*	
C15	0.1444 (2)	0.58901 (10)	0.05096 (15)	0.0603 (5)	
C16	0.2661 (2)	0.55529 (9)	0.10290 (15)	0.0598 (5)	
H16	0.2973	0.5213	0.0629	0.072*	
C17	0.34171 (18)	0.57134 (8)	0.21307 (14)	0.0500 (4)	
H17	0.4236	0.5486	0.2454	0.060*	
C18	0.0653 (3)	0.57206 (13)	−0.07049 (18)	0.0886 (7)	
H18A	0.1121	0.5368	−0.0998	0.133*	
H18B	−0.0290	0.5592	−0.0690	0.133*	
H18C	0.0627	0.6094	−0.1198	0.133*	
C19	0.79899 (16)	0.63248 (8)	0.63886 (12)	0.0439 (4)	
C20	0.81576 (18)	0.56531 (9)	0.64447 (14)	0.0526 (4)	
H20	0.7438	0.5392	0.6596	0.063*	
C21	0.9383 (2)	0.53679 (12)	0.62782 (17)	0.0737 (6)	
H21	0.9476	0.4916	0.6306	0.088*	
C22	1.0455 (2)	0.57422 (17)	0.6073 (2)	0.0898 (8)	
H22	1.1276	0.5547	0.5960	0.108*	
C23	1.0322 (2)	0.64040 (16)	0.60343 (19)	0.0884 (8)	
H23	1.1060	0.6660	0.5902	0.106*	
C24	0.9092 (2)	0.66999 (11)	0.61901 (16)	0.0648 (5)	
H24	0.9011	0.7152	0.6161	0.078*	
C25	0.62860 (15)	0.65060 (7)	0.38786 (12)	0.0388 (3)	
C26	0.62869 (19)	0.71794 (8)	0.37655 (15)	0.0535 (4)	
H26	0.5615	0.7427	0.4020	0.064*	
C27	0.7283 (2)	0.74848 (9)	0.32761 (17)	0.0659 (5)	
H27	0.7285	0.7937	0.3213	0.079*	
C28	0.8269 (2)	0.71204 (11)	0.28830 (16)	0.0674 (5)	
H28	0.8938	0.7326	0.2555	0.081*	
C29	0.8263 (2)	0.64531 (10)	0.29763 (16)	0.0623 (5)	
H29	0.8927	0.6207	0.2708	0.075*	
C30	0.72750 (17)	0.61456 (8)	0.34679 (13)	0.0470 (4)	
H30	0.7274	0.5693	0.3523	0.056*	
N1	0.32254 (14)	0.67817 (6)	0.45484 (11)	0.0485 (3)	
O1	0.39627 (12)	0.54081 (5)	0.60560 (10)	0.0516 (3)	
O2	0.42173 (12)	0.68874 (5)	0.56063 (9)	0.0479 (3)	

### Atomic displacement parameters (Å^2^)

**Table:**

	*U*^11^	*U*^22^	*U*^33^	*U*^12^	*U*^13^	*U*^23^
C1	0.0497 (9)	0.0408 (8)	0.0407 (8)	0.0036 (7)	0.0121 (7)	−0.0042 (6)
C2	0.0716 (12)	0.0627 (11)	0.0474 (9)	−0.0014 (9)	0.0075 (9)	−0.0116 (8)
C3	0.1003 (18)	0.0979 (16)	0.0397 (9)	0.0050 (14)	0.0127 (10)	−0.0130 (9)
C4	0.0994 (18)	0.1110 (18)	0.0431 (10)	0.0056 (15)	0.0299 (11)	0.0040 (10)
C5	0.0721 (13)	0.0765 (12)	0.0525 (10)	0.0011 (10)	0.0297 (9)	0.0065 (9)
C6	0.0502 (9)	0.0455 (8)	0.0424 (8)	0.0034 (7)	0.0163 (7)	0.0007 (6)
C7	0.0387 (8)	0.0359 (7)	0.0450 (8)	0.0019 (6)	0.0143 (6)	−0.0006 (6)
C8	0.0380 (8)	0.0327 (6)	0.0386 (7)	0.0019 (6)	0.0095 (6)	−0.0007 (5)
C9	0.0474 (9)	0.0342 (7)	0.0400 (7)	−0.0052 (6)	0.0092 (6)	−0.0037 (6)
C10	0.0365 (7)	0.0336 (7)	0.0367 (7)	0.0011 (6)	0.0087 (6)	0.0011 (5)
C11	0.0376 (8)	0.0384 (7)	0.0432 (8)	0.0009 (6)	0.0091 (6)	0.0056 (6)
C12	0.0380 (8)	0.0449 (8)	0.0421 (8)	−0.0041 (6)	0.0071 (6)	0.0079 (6)
C13	0.0424 (9)	0.0596 (10)	0.0571 (10)	0.0033 (8)	0.0043 (8)	0.0058 (8)
C14	0.0469 (10)	0.0764 (12)	0.0578 (10)	0.0000 (9)	−0.0045 (8)	0.0142 (9)
C15	0.0549 (11)	0.0763 (12)	0.0443 (9)	−0.0159 (9)	−0.0007 (8)	0.0122 (8)
C16	0.0639 (12)	0.0685 (11)	0.0448 (9)	−0.0030 (9)	0.0077 (8)	−0.0029 (8)
C17	0.0474 (9)	0.0564 (9)	0.0434 (8)	0.0023 (7)	0.0044 (7)	0.0032 (7)
C18	0.0867 (16)	0.1143 (18)	0.0518 (11)	−0.0168 (14)	−0.0127 (11)	0.0055 (11)
C19	0.0387 (8)	0.0588 (9)	0.0328 (7)	−0.0071 (7)	0.0048 (6)	−0.0054 (6)
C20	0.0466 (9)	0.0609 (10)	0.0495 (9)	0.0062 (8)	0.0086 (7)	−0.0033 (7)
C21	0.0593 (13)	0.0976 (15)	0.0604 (12)	0.0261 (12)	0.0052 (10)	−0.0129 (10)
C22	0.0490 (13)	0.153 (2)	0.0647 (13)	0.0147 (15)	0.0076 (10)	−0.0331 (15)
C23	0.0452 (12)	0.158 (3)	0.0641 (13)	−0.0305 (14)	0.0169 (10)	−0.0259 (14)
C24	0.0526 (11)	0.0887 (13)	0.0540 (10)	−0.0245 (10)	0.0137 (8)	−0.0125 (9)
C25	0.0376 (8)	0.0438 (8)	0.0339 (7)	−0.0015 (6)	0.0059 (6)	0.0043 (5)
C26	0.0528 (10)	0.0465 (9)	0.0632 (10)	0.0021 (8)	0.0168 (8)	0.0103 (7)
C27	0.0689 (13)	0.0532 (10)	0.0760 (12)	−0.0082 (9)	0.0166 (10)	0.0216 (9)
C28	0.0603 (12)	0.0833 (14)	0.0628 (11)	−0.0131 (10)	0.0230 (9)	0.0221 (10)
C29	0.0580 (11)	0.0778 (13)	0.0579 (10)	−0.0001 (9)	0.0277 (9)	0.0075 (9)
C30	0.0486 (9)	0.0516 (9)	0.0431 (8)	−0.0007 (7)	0.0153 (7)	0.0022 (6)
N1	0.0471 (8)	0.0489 (7)	0.0481 (7)	0.0095 (6)	0.0079 (6)	0.0031 (6)
O1	0.0489 (7)	0.0466 (6)	0.0612 (7)	−0.0109 (5)	0.0165 (5)	−0.0027 (5)
O2	0.0516 (7)	0.0433 (6)	0.0473 (6)	0.0134 (5)	0.0082 (5)	−0.0046 (4)

### Geometric parameters (Å, º)

**Table:**

C1—C2	1.383 (2)	C15—C18	1.512 (3)
C1—C6	1.387 (2)	C16—C17	1.386 (2)
C1—C9	1.519 (2)	C16—H16	0.9300
C2—C3	1.379 (3)	C17—H17	0.9300
C2—H2	0.9300	C18—H18A	0.9600
C3—C4	1.377 (3)	C18—H18B	0.9600
C3—H3	0.9300	C18—H18C	0.9600
C4—C5	1.386 (3)	C19—C24	1.382 (2)
C4—H4	0.9300	C19—C20	1.387 (2)
C5—C6	1.391 (2)	C20—C21	1.382 (3)
C5—H5	0.9300	C20—H20	0.9300
C6—C7	1.473 (2)	C21—C22	1.360 (4)
C7—O1	1.2093 (17)	C21—H21	0.9300
C7—C8	1.544 (2)	C22—C23	1.363 (4)
C8—O2	1.4730 (17)	C22—H22	0.9300
C8—C10	1.5248 (19)	C23—C24	1.391 (3)
C8—C9	1.5384 (19)	C23—H23	0.9300
C9—C19	1.513 (2)	C24—H24	0.9300
C9—H9	0.9800	C25—C30	1.385 (2)
C10—C25	1.515 (2)	C25—C26	1.387 (2)
C10—C11	1.517 (2)	C26—C27	1.384 (2)
C10—H10	0.9800	C26—H26	0.9300
C11—N1	1.2813 (19)	C27—C28	1.377 (3)
C11—C12	1.467 (2)	C27—H27	0.9300
C12—C17	1.380 (2)	C28—C29	1.373 (3)
C12—C13	1.403 (2)	C28—H28	0.9300
C13—C14	1.374 (2)	C29—C30	1.382 (2)
C13—H13	0.9300	C29—H29	0.9300
C14—C15	1.383 (3)	C30—H30	0.9300
C14—H14	0.9300	N1—O2	1.4171 (16)
C15—C16	1.391 (3)		
			
C2—C1—C6	119.92 (16)	C14—C15—C18	121.76 (19)
C2—C1—C9	127.88 (16)	C16—C15—C18	120.6 (2)
C6—C1—C9	112.18 (12)	C17—C16—C15	121.30 (18)
C3—C2—C1	118.52 (19)	C17—C16—H16	119.4
C3—C2—H2	120.7	C15—C16—H16	119.4
C1—C2—H2	120.7	C12—C17—C16	120.64 (16)
C4—C3—C2	121.51 (19)	C12—C17—H17	119.7
C4—C3—H3	119.2	C16—C17—H17	119.7
C2—C3—H3	119.2	C15—C18—H18A	109.5
C3—C4—C5	120.86 (19)	C15—C18—H18B	109.5
C3—C4—H4	119.6	H18A—C18—H18B	109.5
C5—C4—H4	119.6	C15—C18—H18C	109.5
C4—C5—C6	117.4 (2)	H18A—C18—H18C	109.5
C4—C5—H5	121.3	H18B—C18—H18C	109.5
C6—C5—H5	121.3	C24—C19—C20	118.24 (17)
C1—C6—C5	121.73 (16)	C24—C19—C9	120.75 (16)
C1—C6—C7	108.98 (13)	C20—C19—C9	121.00 (14)
C5—C6—C7	129.29 (16)	C21—C20—C19	120.65 (19)
O1—C7—C6	128.81 (14)	C21—C20—H20	119.7
O1—C7—C8	125.55 (13)	C19—C20—H20	119.7
C6—C7—C8	105.63 (12)	C22—C21—C20	120.5 (2)
O2—C8—C10	104.02 (10)	C22—C21—H21	119.7
O2—C8—C9	106.79 (11)	C20—C21—H21	119.7
C10—C8—C9	123.38 (12)	C21—C22—C23	119.8 (2)
O2—C8—C7	101.00 (11)	C21—C22—H22	120.1
C10—C8—C7	114.55 (11)	C23—C22—H22	120.1
C9—C8—C7	104.63 (11)	C22—C23—C24	120.5 (2)
C19—C9—C1	111.84 (12)	C22—C23—H23	119.7
C19—C9—C8	114.58 (11)	C24—C23—H23	119.7
C1—C9—C8	101.92 (12)	C19—C24—C23	120.2 (2)
C19—C9—H9	109.4	C19—C24—H24	119.9
C1—C9—H9	109.4	C23—C24—H24	119.9
C8—C9—H9	109.4	C30—C25—C26	118.82 (15)
C25—C10—C11	110.78 (11)	C30—C25—C10	120.50 (13)
C25—C10—C8	115.75 (11)	C26—C25—C10	120.67 (14)
C11—C10—C8	98.88 (11)	C27—C26—C25	120.40 (17)
C25—C10—H10	110.3	C27—C26—H26	119.8
C11—C10—H10	110.3	C25—C26—H26	119.8
C8—C10—H10	110.3	C28—C27—C26	120.10 (17)
N1—C11—C12	120.82 (14)	C28—C27—H27	120.0
N1—C11—C10	113.96 (13)	C26—C27—H27	120.0
C12—C11—C10	125.06 (13)	C29—C28—C27	119.91 (17)
C17—C12—C13	118.23 (15)	C29—C28—H28	120.0
C17—C12—C11	121.54 (14)	C27—C28—H28	120.0
C13—C12—C11	120.23 (15)	C28—C29—C30	120.25 (18)
C14—C13—C12	120.46 (18)	C28—C29—H29	119.9
C14—C13—H13	119.8	C30—C29—H29	119.9
C12—C13—H13	119.8	C29—C30—C25	120.50 (16)
C13—C14—C15	121.73 (17)	C29—C30—H30	119.7
C13—C14—H14	119.1	C25—C30—H30	119.7
C15—C14—H14	119.1	C11—N1—O2	109.14 (12)
C14—C15—C16	117.61 (16)	N1—O2—C8	107.42 (10)

### Hydrogen-bond geometry (Å, º)

**Table:**

*D*—H···*A*	*D*—H	H···*A*	*D*···*A*	*D*—H···*A*
C10—H10···O1^i^	0.98	2.47	3.4169 (18)	163
C2—H2···N1^ii^	0.93	2.56	3.280 (2)	135

Symmetry codes: (i) −*x*+1, −*y*+1, −*z*+1; (ii) *x*+1/2, −*y*+3/2, *z*+1/2.

## References

[bb1] Akhazzane, M., Zouihri, H., Daran, J.-C., Kerbal, A. & Al Houari, G. (2010). *Acta Cryst.* E**66**, o3067. 10.1107/S1600536810044387 PMC3011461 21589377

[bb2] Al Houari, G., Baba, M. F., Miqueu, K., Sotiropoulos, J. M., Garrigues, B., Benhadda, T., Benlarbi, N., Safir, I. & Kerbal, A. (2008). *J. Marocain Chim. Heterocycl.* **7**, 16–20.

[bb3] Al Houari, G., Bennani-Kella, A., Bennani, B., Daoudi, M., Benlarbi, N., El Yazidi, M., Garrigues, B. & Kerbal, A. (2010). *J. Marocain Chim. Heterocycl.* **9**, 36–43.

[bb4] Bruker (2009). *APEX2* and *SAINT*. Bruker AXS Inc., Madison, Wisconsin, USA.

[bb5] Farrugia, L. J. (2012). *J. Appl. Cryst.* **45**, 849–854.

[bb6] Sheldrick, G. M. (2015*a*). *Acta Cryst.* A**71**, 3–8.

[bb7] Sheldrick, G. M. (2015*b*). *Acta Cryst.* C**71**, 3–8.

[bb8] Spek, A. L. (2009). *Acta Cryst.* D**65**, 148–155. 10.1107/S090744490804362X PMC2631630 19171970

[bb9] Westrip, S. P. (2010). *J. Appl. Cryst.* **43**, 920–925.

