# Supplementary figures and images for: Crystal structure of 3,4′-diphenyl-3′-p-tolyl-4′H-spiro­[indan-2,5′-[1,2]oxazol]-1-one
Source: Acta Crystallogr E Crystallogr Commun. 2015 Oct 24;71(Pt 11):o873–4. doi: 10.1107/S2056989015019581 (PMC4645049; doi:10.1107/S2056989015019581)

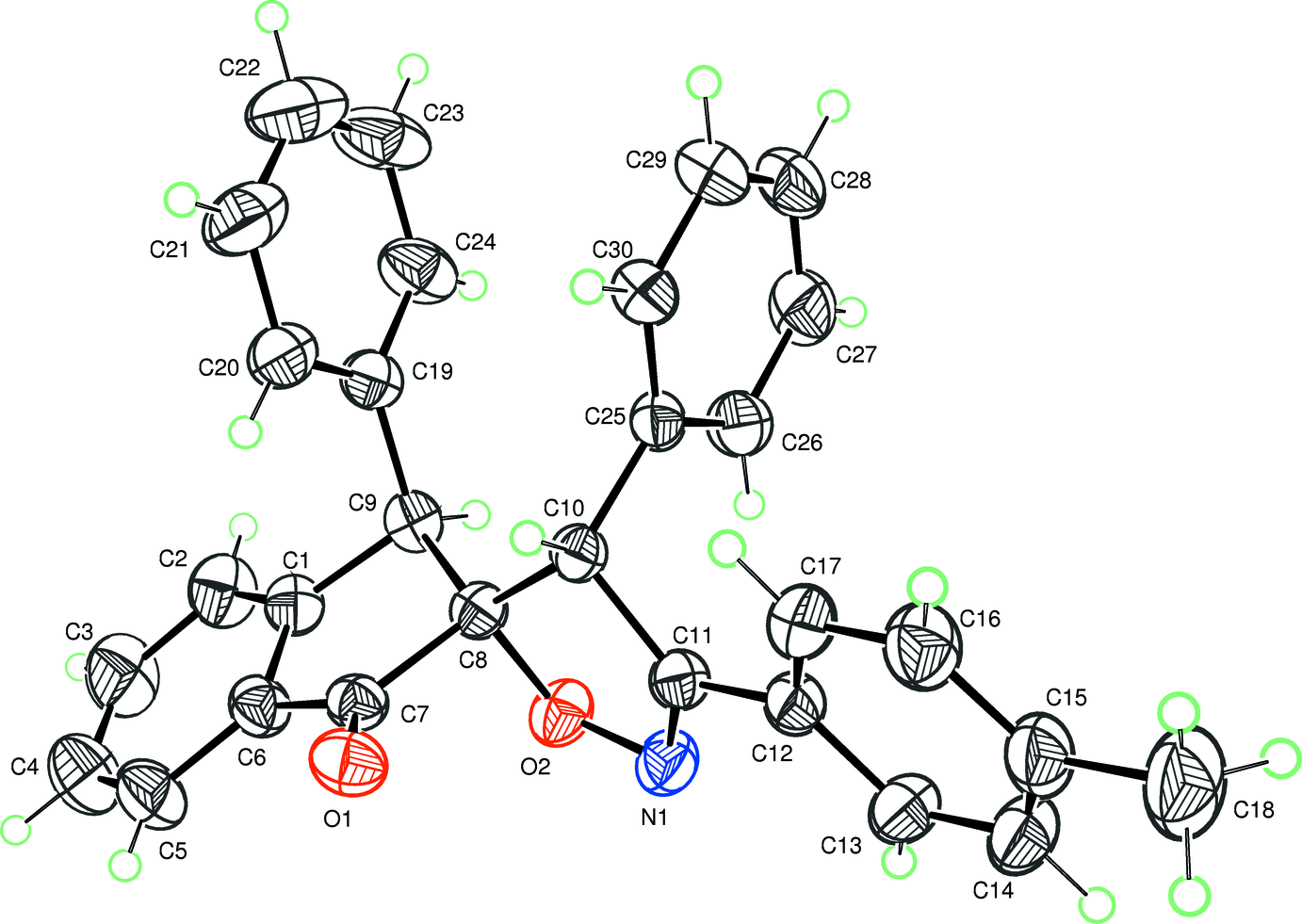

Supplement: Supplementary file 4 [file e-71-0o873-fig1.tif]

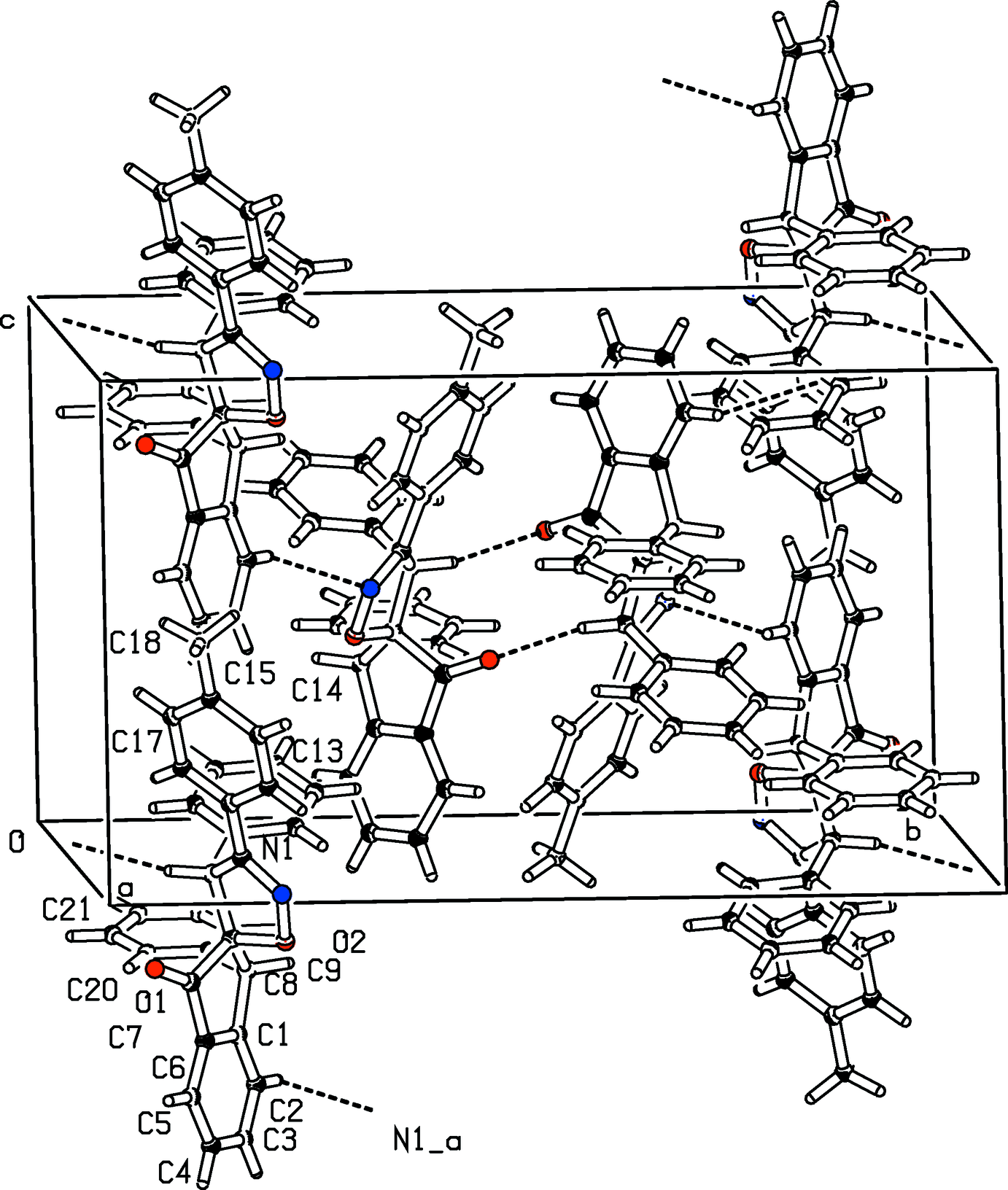

Supplement: Supplementary file 5 [file e-71-0o873-fig2.tif]
